# Supplementary material for: An interplay between cellular growth and atypical fusion defines morphogenesis of a modular glial niche in Drosophila
Source: Nat Commun. 2022 Aug 25;13:4999. doi: 10.1038/s41467-022-32685-3 (PMC9411534; doi:10.1038/s41467-022-32685-3)

Supplementary information

An interplay between cellular growth and atypical fusion defines morphogenesis of a modular glial niche in Drosophila

Maria Alexandra Rujano, David Briand, Bojana Đelić, Julie Marc and Pauline Spéder

The supplementary information contains:

- Supplementary movies (14)
- Supplementary tables (2)
- Legends of Supplementary figures
- Supplementary references
- Supplementary figures (10)

Supplementary Table 1. *Drosophila* transgenic lines used in this study.

Strains	Source	Stock number/Reference
w^{1118}	BDSC	5905
Nervana2::GFP (Nrv2::GFP)	BDSC	6828
tubulin-GAL80$^{thermosensitive(ts)}$	BDSC	65406
Cre recombinase	BDSC	851
yw, hs-FLP	Andrea Brand lab	
CoinFLP	BDSC	58750
cyp4g15-GAL4	BDSC	39103
cyp4g15-FRT-STOP-FRT-LexA	This study	
cyp4g15-FLP	This study	
cyp4g15-QF2	This study	
cyp4g15-mtd::Tomato	This study	
alrm-GAL4	Marc Freeman lab	[1]
mbc-GAL4 (Trojan)	BDSC	66840
UAS-H2B::YFP (Hist::YFP)	François Schweisguth lab	[2]
UAS-H2B::RFP (Hist::RFP)	Yohanns Bellaïche lab	[3]
UAS-His3.3.mIFP-T2A-HO1 (Hist::IFP)	BDSC	64184
UAS-GFP	BDSC	1522
UAS-mCD8::GFP	BDSC	5130
UAS-mCD8::RFP	BDSC	27399
UAS-mito::HA::GFP	BDSC	8443
UAS-GFP::Alix	Jean-René Huynh lab	[4]
UAS-hβactin::ECFP	BDSC	7064
LexAOp-mCherry::mito.OMM	BDSC	66531
UAS-Raeppli CAAX 43E	BDSC, This study	55082
UAS-Raeppli NLS 53D	BDSC, This study	55087
LexAop-Raeppli CAAX 43E	BDSC, This study	55082
UAS-mRFP::Scra	BDSC	52220
QUAS-NLS-LacZ	BDSC	30006
Fly FUCCI	BDSC	55117
G-TRACE	BDSC	28280
iTRACE	BDSC	66387
Ubi-p63E-GFP::Pavarotti	David Glover lab	[5]
UAS-mbc RNAi	BDSC	32355
UAS-WASp RNAI	BDSC	51802
UAS-rst RNAi	VDRC	27223
UAS-hbs RNAi	BDSC	57003
UAS-kirre RNAi	VDRC	27227
UAS-lmd RNAi	BDSC	42871
UAS-sns RNAi	BDSC	64872
UAS-dock RNAi	BDSC	27728
UAS-dup RNAi	BDSC	29562
UAS-stg RNAi	BDSC	34831
UAS-Kaede	BDSC	26161
UAS-Δp60		[6]

Supplementary Table 2. Primers used for generating *Drosophila* transgenic lines used in this study.

Purpose	# Fragments	primer name and 5' to 3' sequence
**cyp4g15 enhancer fused to hsp70 minimal promoter**	Fragment 1	Forward primer:cyp4g15eh attb1<br>ggggacaagtttgtacaaaaaagcaggctctgcgaggcaaccgtggagattctattggg
		Reverse primer: cyp4g15-hsp70 up<br>gaagcgcctctatttatactccggcgctccctttcagtgcagagactcgtatctggatttg
	Fragment 2	Forward primer: cyp4g15-hsp70 down<br>caaatccagatacgagtctctgcactgaaagggagcgccggagtataaatagaggcgcttc
		Reverse primer: hsp70 attb5r<br>ggggacaacttttgtatacaaagttgttattcagagttctcttcttgtattcaataattac

**cyp4g15 enhancer fused to DSCP minimal promoter**	Fragment 1	Forward primer: cyp4g15eh attb1<br>ggggacaagtttgtacaaaaaagcaggctctgcgaggcaaccgtggagattctattggg
		Reverse primer: cyp4g15-dscp up<br>gcgctcgatccccgggcgagctcgcctttcagtgcagagactcgtatctgga
	Fragment 2	Forward primer: cyp4g15-dscp down<br>caaatccagatacgagtctctgcactgaaaggcgagctcgcccggggatcgagcgcag
		Reverse primer: dscp attbr5<br>ggggacaacttttgtatacaaagttgtgtttggtatgcgtcttgtgattcaaagttggc

**FLP construct driven by cyp4g15^{DSCP}**		Forward primer: Flp attb5<br>ggggacaactttgtatacaaaagttggcatgccacaatttggtatattatgtaaaacacca
		Reverse primer: Flp attb2<br>ggggaccactttgtacaagaaagctgggtattatatgcgtctatttatgtaggatgaaaggta

**mtd-Tomato construct driven by cypAg15^{DSCP}**		Forward primer: mtd-tomato attb5<br>ggggacaactttgtatacaaaagttggcatgggttgctgtttctccaagaccatg
		Reverse primer: mtd-tomato attb2<br>ggggaccactttgtacaagaaagctgggtattaagcgtaatctggaacgtcatatgg

**FRT-STOP-FRT-LexA construct driven by cypAg15^{DSCP}**	Fragment 1	Forward primer: attb5 frt<br>ggggacaactttgtatacaaaagttggcgcatgcctgcaggtcccctc
		Reverse primer: frt-lexa up<br>gttgtcgggccgtgagagccttcatggtgacactatagaatacgaattggtc
	Fragment 2	Forward primer: frt-lexa down<br>gaccaattcgtattctatagtgtcaccatgaaggctctcacggcccgacaac
		Reverse primer: lexa attb2<br>ggggaccactttgtacaagaaagctgggtattaggagcttatctggctcagcaaagcgg

Supplementary Figure 1. Cortex glia territory in the *Drosophila* CNS.

a) Upper panels show a side view of the CNS showing the astrocyte glia (*alrm>CD8::RFP*, magenta) compartment compared to Nrv2::GFP (green) expression (upper panels). Lower panels are side views of a CNS expressing membrane targeted CD8 (*mCD8::GFP*, green) and Histone (*Hist::RFP*, magenta) in the CG (*cyp4g15-GAL4*). Note that CG are ventral in the VNC, paralleling NSC location, while the astrocyte glia are restricted to the dorsal side. Scale bars: 50 μm.

Supplementary Figure 2. Cortex glia growth over time.

a) Schematics of the Raeppli multicolour lineage tracing tool. Adapted from [7]. The Raeppli construct contains 5X UAS and LexO sites that act on a basal *hsp70* promoter (cyan ellipse) for expression of chosen fluorescent proteins. UAS and LexO sites are flanked by Lox2272 or LoxP sites, respectively. Cre protein excises one of the enhancers in a mutually exclusive manner. A single attB site (light grey arrow) downstream of the enhancer and promoter, is available for recombination with one of the attP sites (dark grey arrows). A full *hsp70* promoter (dark blue ellipse) regulates the expression of the integrase gene which is blocked by the presence of a stop cassette (black box) flanked by FRT sites (purple triangles). Fluorescent protein genes have a stop codon and are arranged in a linear fashion each preceded by an attP site. Expression of the flippase (Flp) removes the stop cassette and triggers expression of integrase. Heat shock then causes the expression of the integrase gene and the Integrase protein recombines the attB site with one of the attP sites of the construct and removes the in between region. The remaining fluorescent protein is then expressed by the action of LexA or Gal4. Since each fluorescent protein gene is followed by a stop codon, the only expressed fluorescent protein is the one brought closest to the promoter.

b) Quantification shown as dot plot of the number of Raeppli-CAAX clones per VNC without induction, observed at ALH72. N = 11 VNCs. No induction results in very few CG clones, all displaying only one colour.

c) Progressive increase in CG nuclei numbers visualized at ALH0, ALH24, ALH48, ALH72 and ALH96 (at 25°C). CG nuclei are labelled with *Hist::RFP* driven by *cyp4g15-GAL4*. Scale bars: 50 μm.

d) Quantification of CG nuclei number in the CNS at ALH0 (n=8), ALH24 (n=11), ALH48 (n=8), ALH72 (n=4) and ALH96 (n=7). n, number of CNS. Results are presented as box and whisker plots. Data statistics: ordinary one-way ANOVA with a Tukey's multiple comparison test.

e, f) Quantification of CG nuclei in Raeppli clones induced in CG before or at ALH0 (e) and assessed at ALH0 (n=43), ALH24 (n=9), ALH48 (n=76), ALH72 (n=46) and ALH96 (n=42) at 25°C. n, number of clones. The pan-glial marker Repo (magenta) was used to identify the CG nuclei within clones. Scale bars: 50 µm. The quantification (f) was performed in the brighter clones (mTFP1, cyan) to facilitate earlier time points assessment. The inset at ALH48 shows close-up of such clones and their contained nuclei. Results are presented as box and whisker plots. Data statistics: ordinary one-way ANOVA with a Tukey's multiple comparison test.

g) Visualisation of neighbouring Raeppli clones using the nuclear targeted Raeppli constructs at ALH24, ALH48, ALH72 and ALH96 at 25°C, with induction through heat shock performed at ALH0-2. Scale bars: 50 µm.

h) Analysis of CG behaviour by multicolour lineage tracing using Raeppli-CAAX (membrane). Hs-Flp and heat shock induction at 37°C were performed at ALH62, and resulting clones were visualised at ALH96 (all development at 25°C). Selected z-plane of a VNC. Scale bars: 20 µm.

Source data are provided as a Source Data file.

Supplementary Figure 3. Multiple proliferative strategies fuel cortex glia growth.

a) Schematics of the Drosophila Fucci tool, adapted from [8]. The colours used in our scheme, reflect the colours used in Fig. 2a,b and 3b. In early M phase, both GFP-E2F1$_{1–230}$ (green) and mRFP1-CycB$_{1–266}$ (magenta) are present thus labelling the cells grey. Midway mitotic stage, the APC/C marks mRFP1-CycB$_{1–266}$ for proteasomal degradation leaving the cells fluorescing green due to GFP-E2F1$_{1–230}$ expression. As cells progress from G1 to S phase, CRL4^{Cdt2} degrades GFP-E2F1$_{1–230}$, and cells are thus labelled in magenta, because only mRFP1-CycB$_{1–266}$ is present. After cells enter G2 phase, GFP-E2F1$_{1–230}$ protein levels reaccumulate, marking the cells grey due to the presence of mRFP1-CycB$_{1–266}$.

b) Plot of nuclear volume against total FISH counts for chromosome 2 and 3 in CG, all timepoints (ALH0-ALH96, n = 602) mixed. n, number of CG nuclei.

c) Still images of a time-lapse movie (Supp. movie 4) of mitotic CG expressing Hist::RFP (magenta) to label nuclei and Lamin::GFP (green) to label the nuclear envelope. Scale bar: 5 µm.

d) Expression of mRFP::scra (magenta) in CG to monitor midbodies along CG membranes. CG membranes and nuclei are labelled with Nrv2::GFP (green) and Hist::IFP (blue) respectively. Arrows indicate midbodies. Scale bar: 10 µm.

e) Representative pictures of larval VNCs expressing Hist::RFP in CG (magenta) and stained with Drosophila cleaved caspase 1 (Dcp-1, green) at ALH24, ALH48 and ALH72, in

control CNS and in CNS where CG-specific downregulation of doubled-parked (*dup RNAi*) was induced. Scale bar: 20 μm. Apoptotic CG nuclei are visualised in grey.
f) Close-up of apoptotic nuclei (grey) from CG> *dup RNAi* at ALH48. Hist::RFP (magenta); Dcp-1 (green). Scale bar 1 μm.
Source data are provided as a Source Data file.

Supplementary Figure 4. Cortex glia cells form syncytia connected by cytoplasmic bridges.
a) Puncta containing both anillin (mRFP::scra, magenta) and Mucin-D (anti-Mucin-D, cyan), are localising along the CG membrane (Nrv2::GFP, green). CG nuclei are stained with His::IFP (*CG>His::IFP*, grey). Scale bars: 2 μm.
b) Puncta enriched both in Mucin-D (anti-Mucin-D, grey) and Pavarotti (Ubi-p63E-GFP::Pavarotti, green), two classical components of midbodies and intercellular bridges, are found along the CG membrane (*cyp4g15-mtd::Tomato*, magenta). Scale bars: 2 μm.
c) CG connection via the midbodies marked by anillin (mRFP::scra, magenta) assessed by FLIP of cytosolic GFP (green). Top panels depict a region in the VNC before (pre-bleach) and after bleaching (post-bleach). CG nuclei are labelled with *Hist::RFP* (magenta). The bleached area delineated by the white dashed square is placed close to an isolated midbody (clear blue inset) in between CG cells. Bottom panels show intermediate time points (GFP only, pseudocolored with thermal LUT) during continuous photobleaching. Scale bars: 10 μm.
d) Selected slices (z11, left panel and z40, right panel) from Z-stacks before and after photoconversion shown in Fig. 4g are displayed next to each other. The cKaede signal overlaps with several midbodies-like (mRFP::scra puncta, dashed white circles) throughout the Z-stack, showing that CG units are rich in intercellular bridges. Scale bar: 10 μm. z-step is 0.50 μm.

Supplementary Figure 5. Cortex glia undergo homotypic cell-cell fusion.
a) Quantification of the number of Raeppli clones at ALH24 (n=5), ALH48 (n=12), ALH72 (n=9) and ALH96 (n=9) at 25°C. n, number of CNS analysed. Results are presented as box and whisker plots. Data statistics: ordinary one-way ANOVA with a Tukey's multiple comparison test.
b) Quantification of colour overlap events in Raeppli clones at ALH24 (n=5), ALH48 (n=12), ALH72 (n=9) and ALH96 (n=9) at 25°C. n, number of CNS analysed. Results are presented

as box and whisker plots. Data statistics: ordinary one-way ANOVA with a Tukey's multiple comparison test.

c) Schematics of the Drosophila Coin-FLP technique adapted from [9]. *CoinFLP-Gal4* used with a *FLP*-expressing enhancer triggers the recombination between either the canonical *FRT* sites, resulting in excision of the STOP cassette and expression of LexGAD, or between the *FRT3* sites, that results in excision of the stop cassette and Lex-GAD, thus triggering Gal4 expression.

d-e) Mitochondrial exchange between CG units assessed in Coin-FLP clones. Clones expressing mitochondrial markers Mito::GFP (green) or mCherry::Mito (magenta), show no overlap (d) or complete overlap (e, likely due to polyploidy). Scale bar: 5 µm.

f) Higher magnification of neighbouring nuclear Raeppli clones that share nuclear material. ALH48 at 25°C. Scale bar: 5 µm.

g) Continuity between CG units due to cellular fusion was assessed by photoconversion of cytosolic Kaede expressed in the CG in combination with early induction of multicolour labelling of CG nuclei (Raeppli-NLS) that leads to clonal labelling of the nuclei in CG units. Iterative photoconversion was performed in a small area (dashed rectangle) within a Raeppli-NLS CG clone containing nuclei of one colour. Top panels depict the assessed area before (pre-photoconversion) and after photoconversion (post-photoconversion). Bottom panels show the converted form (cKaede) only, pseudocolored with thermal LUT before and after photoconversion, with nuclei represented by black discs outlined in the respective Raeppli colour. In total, two different colours of nuclei are joined by the cKaede signal. Scale bars: 10 µm.

Source data are provided as a Source Data file.

Supplementary Figure 6. Assessing the continuity of cytoplasmic compartments by FLIP.

a) Different zones defined by different combinations of GFP and mCherry levels as well as by positions are identified in the area imaged in Fig. 6a-d. Left panel, borders of the zones drawn on a still image of experiment of Fig. 6a (Supp. movie 8) at T0. Right panel, schematic of the area overlaid with the borders and names of the different zones.

b) Estimation of the values that %FL should take to be significantly due to the FLIP experiment rather than chance. For each movie, we performed a Monte-Carlo analysis on %FL in the channel corresponding to the unbleached fluorophore (Fig. 6a: mCherry; 6b: GFP; 6c: GFP and 6d: mCherry). This was achieved by sampling %FL (percentage of change in mean intensity) over ten thousand randomly positioned 10 X 10 µm squares.

Histograms (left panels) represent the distribution of %FL amongst the 10.000 random squares. Cumulative Distribution Functions (middle panels) were generated from the results and used to calculate the %FL value required for a 95% confidence level (right panels).
c) Table of the changes in mean intensities (I_{MEAN}) in GFP and mCherry in the different zones of interest between T0 (start of the movie, frame 0) and T100 (end of the movie/photobleaching, frame 100), and the corresponding %FL values. The significant %FL values take the colour (green or magenta) of the bleached fluorophores, and the ones outside of the zone targeted by the bleach are in a pale yellow background. See Methods for details on the significance.

Supplementary Figure 7. Results of the assessment of cortex glia compartmental continuity by FLIP.
a-d) Individual GFP and mCherry channels before (pre-bleach) and after bleaching (post-bleach), corresponding to experiments shown in Fig. 6a-d respectively. Dashed lines indicate the zones outside of the region that was targeted by the FLIP in which a significant %FL occurred. Scale bars: 10 μm.

Supplementary Figure 8. Cortex glia display cellular hallmarks of cell-cell fusion.
a) Still images of time-lapse movies from three different VNCs (ALH70 at 25°C) expressing a fusion of human β-actin with ECFP in the CG only (*cyp>β-actin::ECFP*, grey). Yellow arrows indicate area of high actin remodelling. Scale bars: 10 μm.

Supplementary Figure 9. Cortex glia rely on classical cell-cell fusion pathways.
a) Schematics of cell-cell fusion based on the model of myofibers formation in Drosophila, adapted from [10–12]. In this model, a fusion-competent cell and a founder cell recognize and bind to each other, creating a so-called fusogenic synapse. Key, well-characterized players for this step are the cell recognition and adhesion molecules that mediate the binding between the two membranes. These molecules are differentially localised in the fusing cells, expressed either by the fusion competent cell (Sns and Hbs) or by the founder cell (Kirre/Duf and Rst). Binding between partners initiate intracellular signalling, through adapter proteins that will lead to remodelling of the actin cytoskeleton in both cells. In the fusion competent cell, the combined actions of multiple actin regulators (WASp, Rac, Scar, Arp2/3) generate invasive podosome-like protrusions at the interface with the founder cell. These structures trigger a Myosin II- and spectrin-mediated response in the founder cell, which is followed by

hemifusion of membranes, pore formation and expansion, culminating in the creation of a multinucleated cell.

b, c) Expression of the lineage tracing tools i-TRACE (b) and G-TRACE (c) under *mbc-GAL4* (Trojan line) to assess *mbc* expression in the CG throughout development. Images were taken at ALH72. Scale bars: 50 µm. Lower panels are higher magnifications of the regions in the dashed squares. Scale bars: 10 µm.

d) RNAi knockdown of cell-cell fusion related genes *kirre* and *lmd* in multicoloured labelled CG in the VNC. RNAi expression was induced at ALH0, larvae were maintained at 29°C and dissected at ALH72. Scale bars: 50 µm.

e, f) Quantification of the number of fusion events per clone (e) and number of clones (f) in multicoloured labelled Raeppli CG clones at ALH72 (at 29°C) after *kirre* and *lmd* knockdown in CG. Ctrl, n=18; *kirre RNAi* n=11; and *lmd RNAi* n=10. n, number of VNCs analysed. Results are presented as box and whisker plots. Data statistics: one-way ANOVA with a Kruskal–Wallis multiple comparison test.

Source data are provided as a Source Data file.

Supplementary Figure 10. Importance of diverse cellular functions on the integrity of the cortex glia network.

a) Effect of overexpression of ∆p60 (blocking PI3K/Akt-dependent growth) and of RNAi knockdown of *stg* (blocking entry in the cell cyle) on CG network architecture (visualised with Nrv2::GFP, portion of the VNC). All ALH72 at 29°C. Scale bar: 10 µm.

b) Effect of down regulation of cell-cell fusion gene *mbc*, *kirre*, and *lmd* on CG network architecture (portion of the VNC). CG network architecture is visualised with Nrv2::GFP. All ALH72 at 29°C. Pink arrows indicate local accumulation of CG membrane. Scale bar: 10 µm.

Supplementary references

1. Doherty, J., Logan, M. a, Taşdemir, O. E. & Freeman, M. R. Ensheathing glia function as phagocytes in the adult Drosophila brain. *J. Neurosci.* **29**, 4768–81 (2009).
2. Bellaïche, Y., Gho, M., Kaltschmidt, J. A., Brand, A. H. & Schweisguth, F. Frizzled regulates localization of cell-fate determinants and mitotic spindle rotation during asymmetric cell division. *Nat. Cell Biol.* **3**, 50–57 (2001).
3. Langevin, J. *et al.* Lethal giant larvae controls the localization of Notch-signaling regulators Numb, neuralized, and Sanpodo in Drosophila sensory-organ precursor cells. *Curr. Biol.* **15**, 955–962 (2005).
4. Eikenes, Å. H. *et al.* ALIX and ESCRT-III Coordinately Control Cytokinetic Abscission during Germline Stem Cell Division In Vivo. *PLOS Genet.* **11**, e1004904 (2015).
5. Minestrini, G., Máthé, E. & Glover, D. M. Domains of the pavarotti kinesin-like protein that direct its subcellular distribution: Effects of mislocalisation on the tubulin and actin cytoskeleton during Drosophila oogenesis. *J. Cell Sci.* **115**, 725–736 (2002).
6. Weinkove, D., Neufeld, T. P., Twardzik, T., Waterfield, M. D. & Leevers, S. J. Regulation of imaginal disc cell size, cell number and organ size by Drosophila class I(A) phosphoinositide 3-kinase and its adaptor. *Curr. Biol.* **9**, 1019–1029 (1999).
7. Kanca, O., Caussinus, E., Denes, A. S., Percival-Smith, A. & Affolter, M. Raeppli: a whole-tissue labeling tool for live imaging of Drosophila development. *Development* **141**, 472–480 (2014).
8. Zielke, N. *et al.* Fly-FUCCI: A Versatile Tool for Studying Cell Proliferation in Complex Tissues. *Cell Rep.* **7**, 588–598 (2014).
9. Bosch, J. A., Tran, N. H. & Hariharan, I. K. CoinFLP: a system for efficient mosaic screening and for visualizing clonal boundaries in Drosophila. *Development* **142**, 597–606 (2015).
10. Chen, E. H. *Invasive Podosomes and Myoblast Fusion*. *Current Topics in Membranes* **68**, (Academic Press Inc., 2011).
11. Deng, S., Azevedo, M. & Baylies, M. Acting on identity: Myoblast fusion and the formation of the syncytial muscle fiber. *Seminars in Cell and Developmental Biology* **72**, 45–55 (2017).
12. Kim, J. H. & Chen, E. H. The fusogenic synapse at a glance. *Journal of cell science* **132**, (2019).

Rujano et al. Supplementary Figure 1

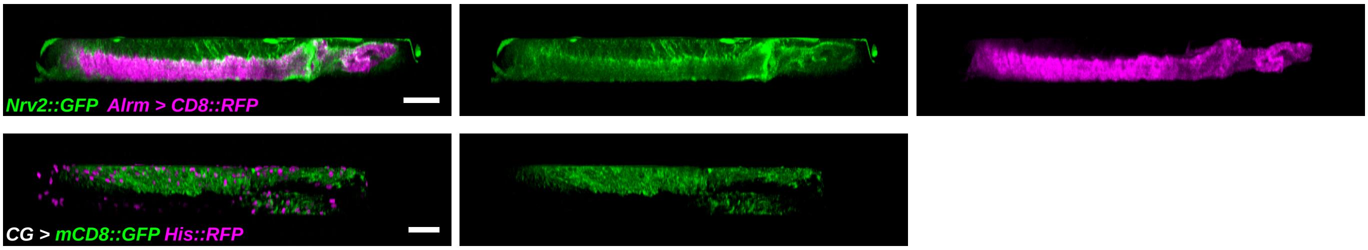

Rujano et al. Supplementary Figure 2

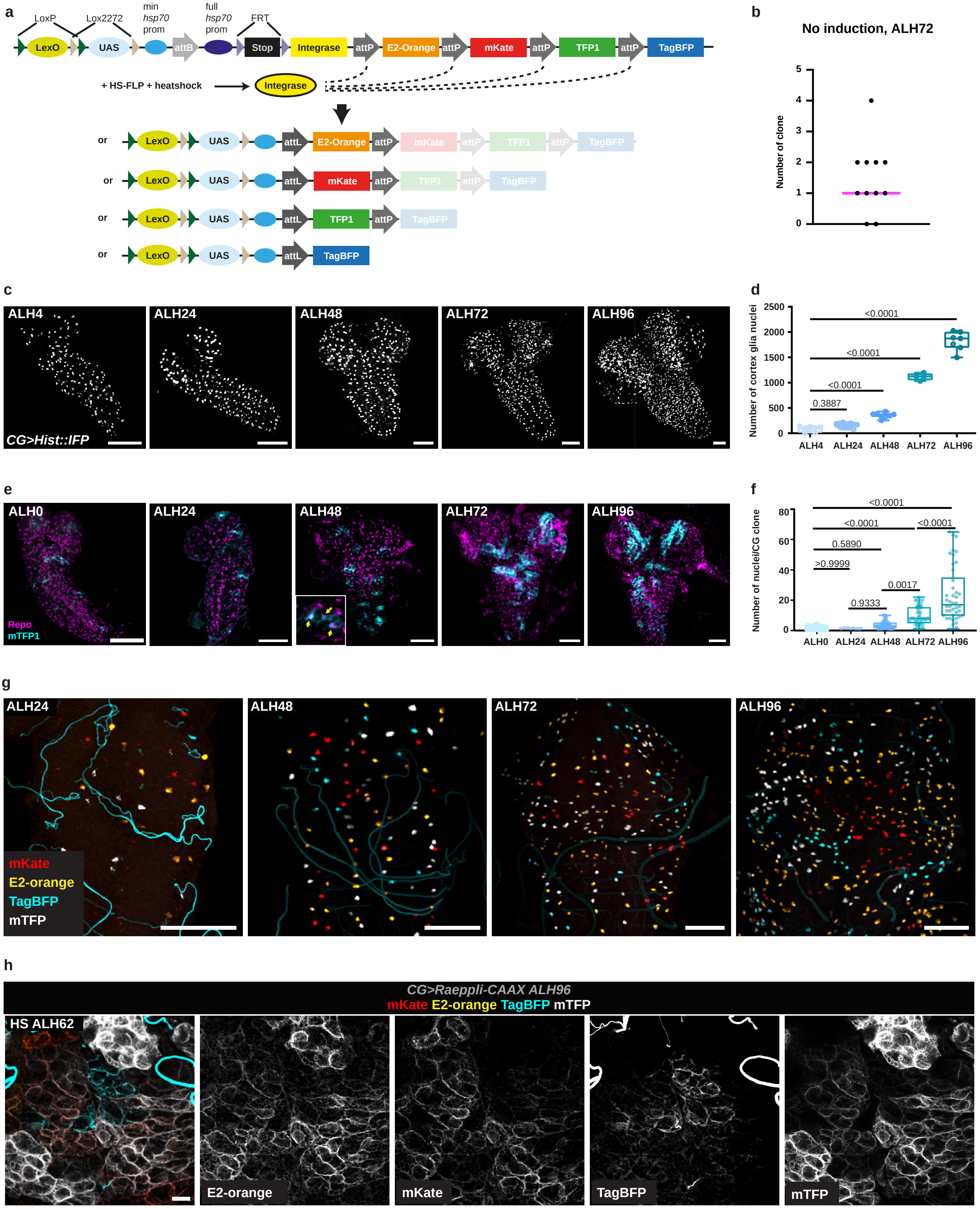

Rujano et al. Supplementary Figure 3

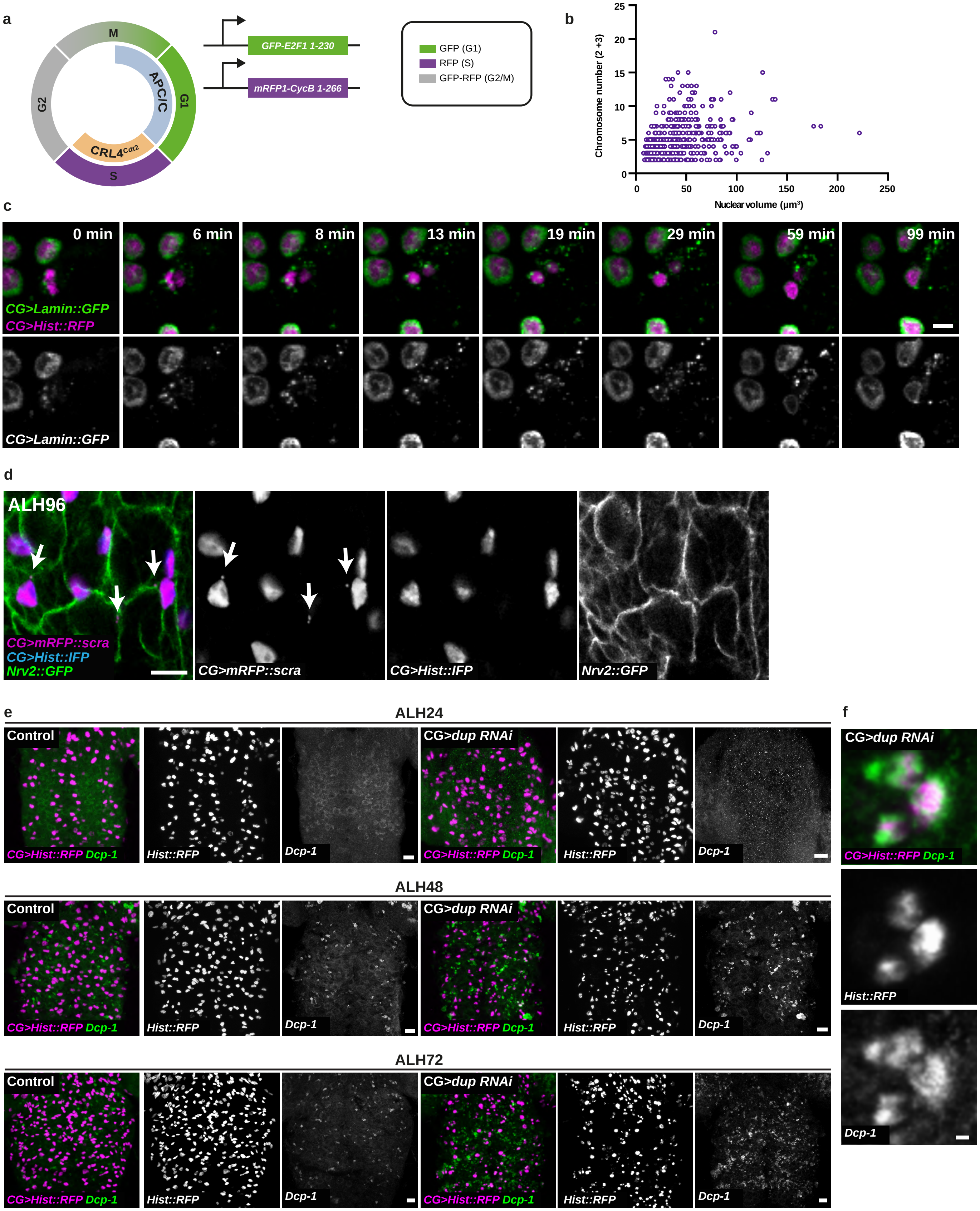

Rujano et al. Supplementary Figure 4

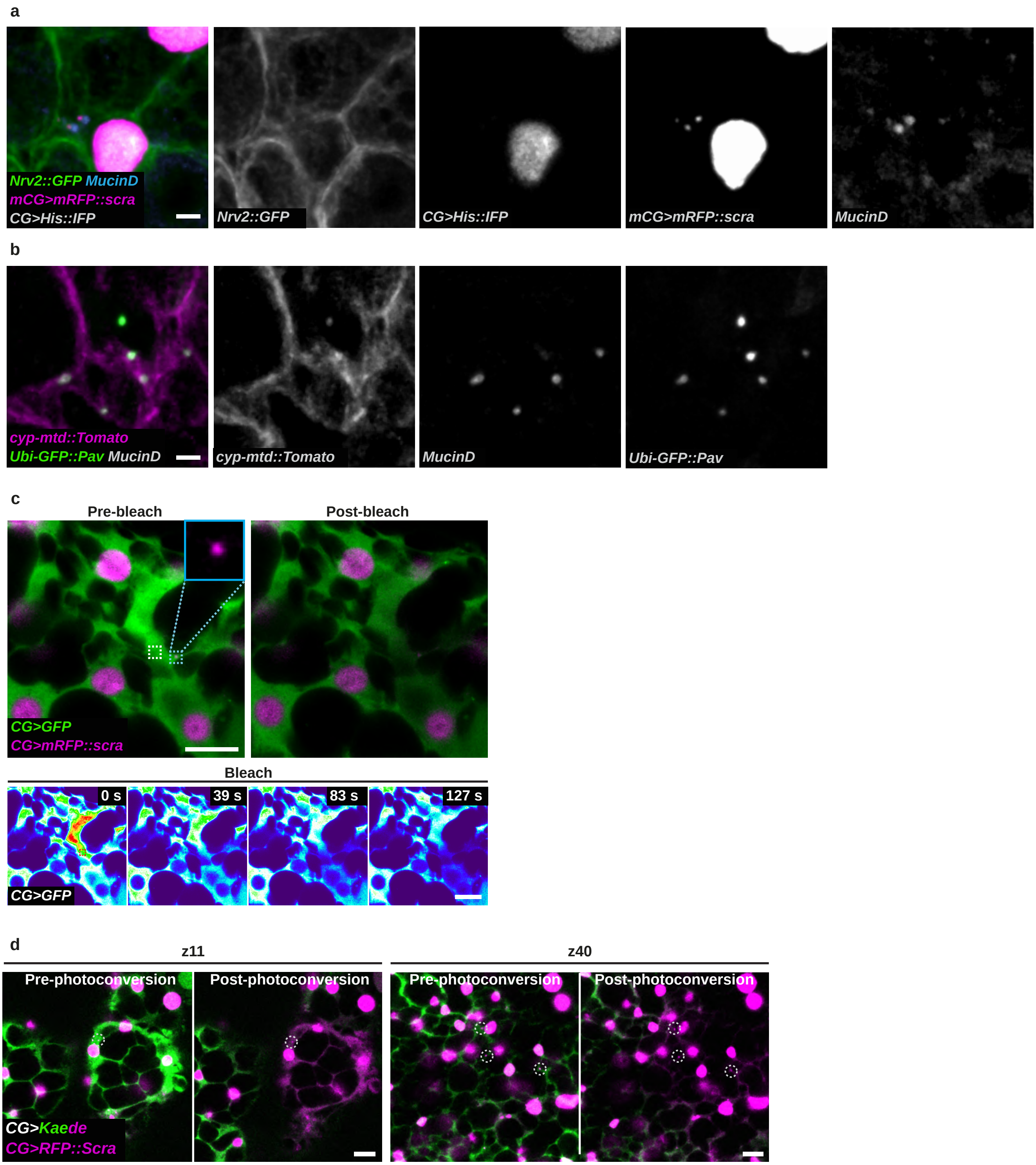

Rujano et al. Supplementary Figure 5

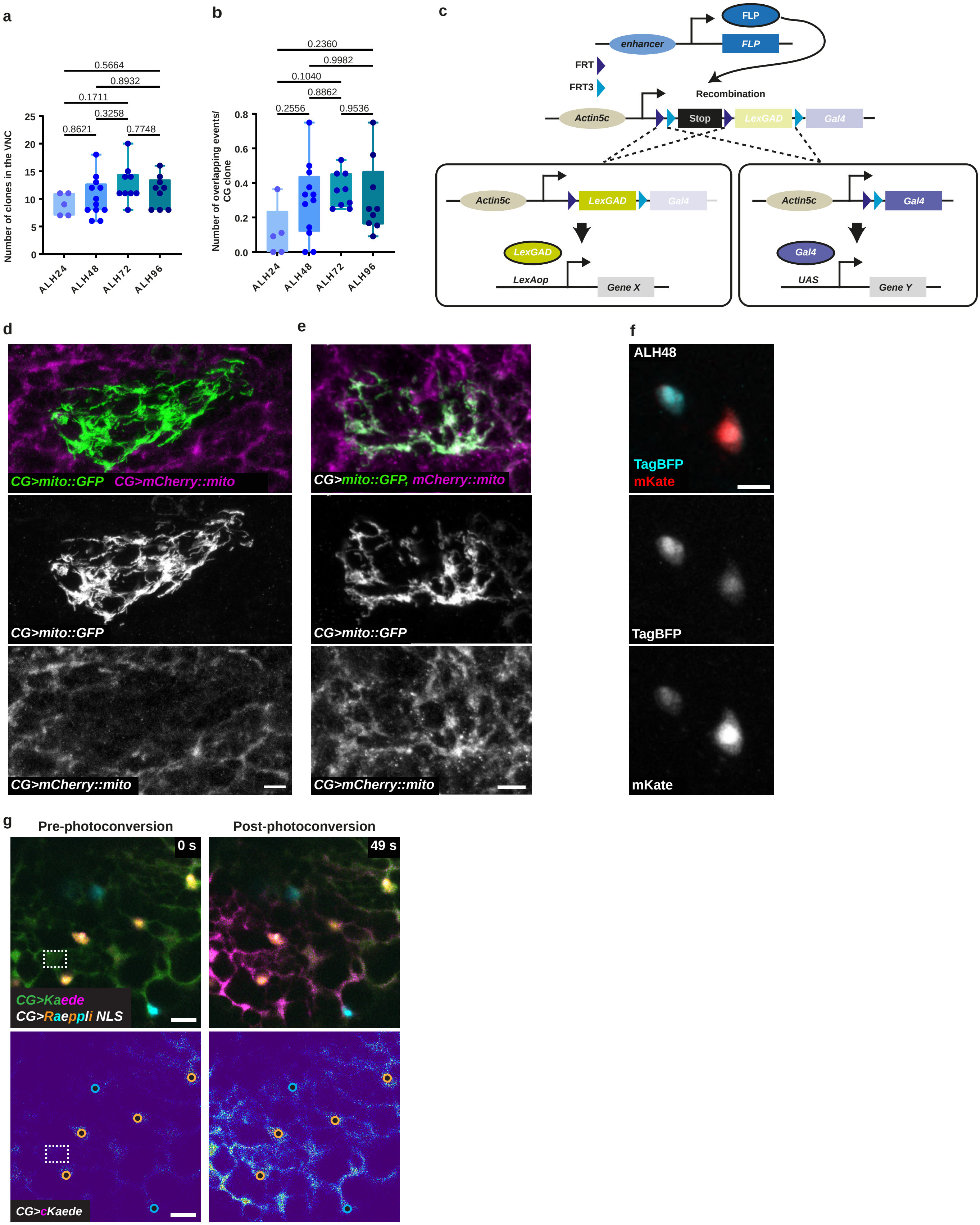

Rujano et al. Supplementary Figure 6

a

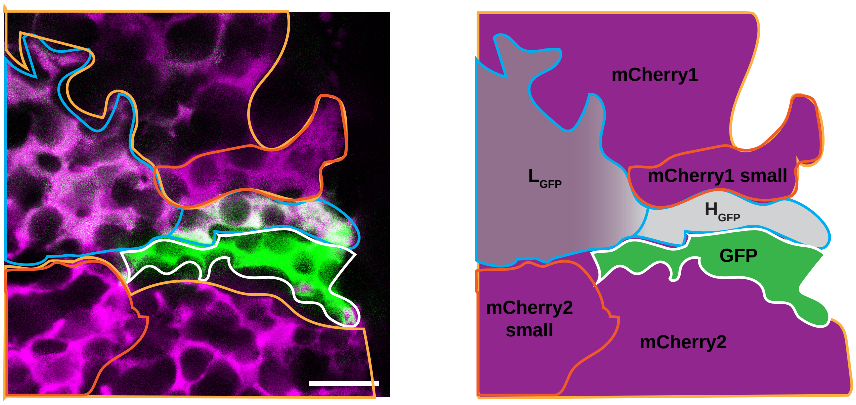

b

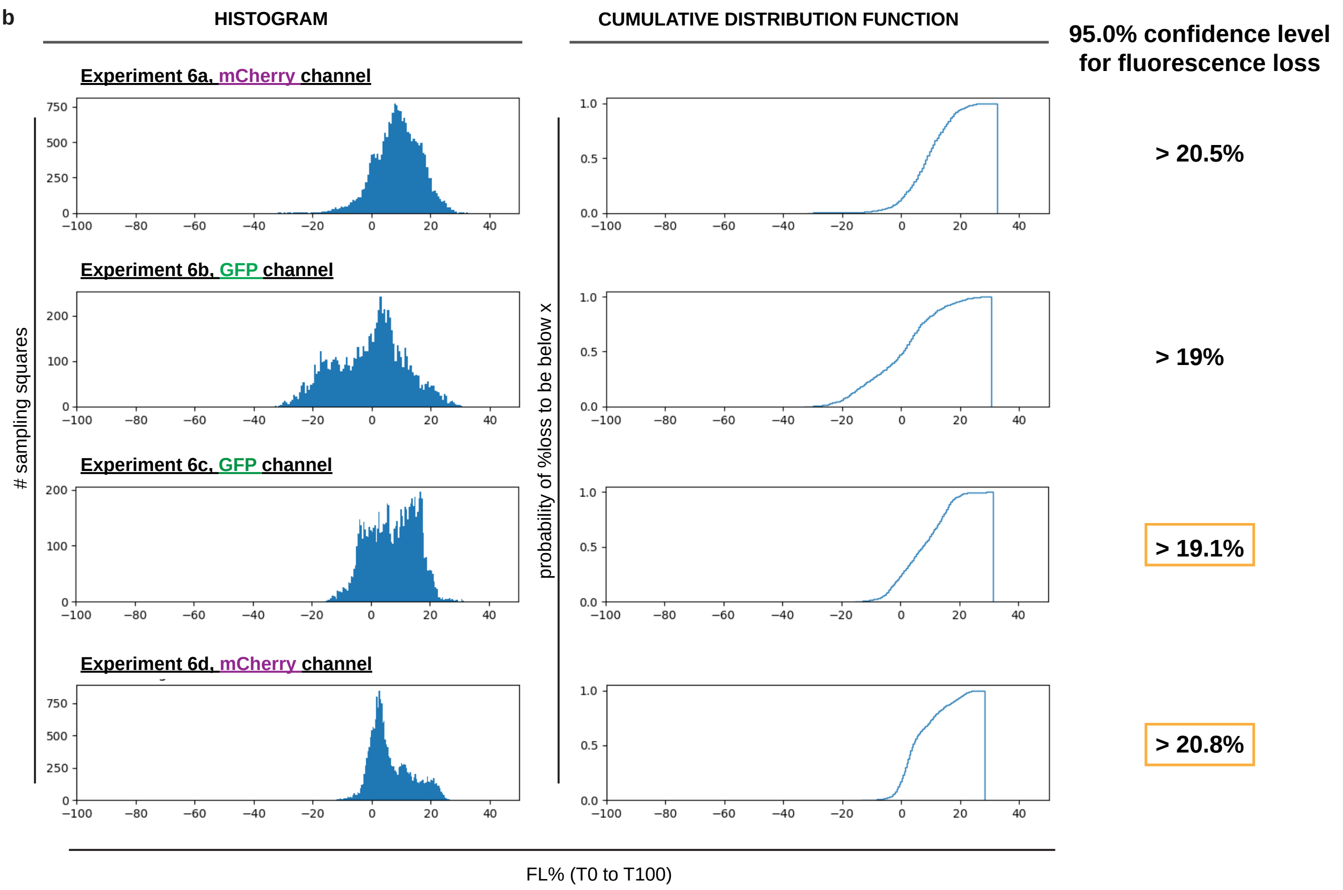

c

	Zone	GFP	mCherry1	mCherry1 small	mCherry 2	mCherry 2 small	H_{GFP}		L_{GFP}	
	Fluorophore	GFP	mCherry	mCherry	mCherry	mCherry	GFP	mCherry	GFP	mCherry
6a	IMEAN T0	132.02	54.43	70.54	68.86	77.77	88.13	80.27	17.46	68.91
	IMEAN T100	17.7	53.04	66.44	60.65	69.76	48.85	74.81	15.36	61.09
	% FL	86.59	2.55	5.81	11.92	10.30	44.57	6.80	12.03	11.35
6b	IMEAN T0	18.98	60.99	71.69	56.02	71.19	41.84	73.58	16.79	63.63
	IMEAN T100	15.64	52.79	57.71	55.23	70.72	35.94	15.87	17.07	63.35
	% FL	17.60	13.44	19.50	1.41	0.66	14.10	78.43	-1.67	0.44
6c	IMEAN T0	19.22	50.81	55.13	56.78	71.83	44.96	27.56	19.95	74.01
	IMEAN T100	16.23	47.96	51.31	46.46	56.2	40.16	25.29	19.32	22.2
	% FL	15.56	5.61	6.93	18.18	21.76	10.68	8.24	3.16	70.00
6d	IMEAN T0	18.85	53.19	51.28	47.57	56.06	43.25	29.5	20.06	25.3
	IMEAN T100	12.97	50.02	49.62	47.02	55.43	39.44	27.77	7.3	21.66
	% FL	31.19	5.96	3.24	1.16	1.12	8.81	5.86	63.61	14.39

Rujano et al. Supplementary Figure 7

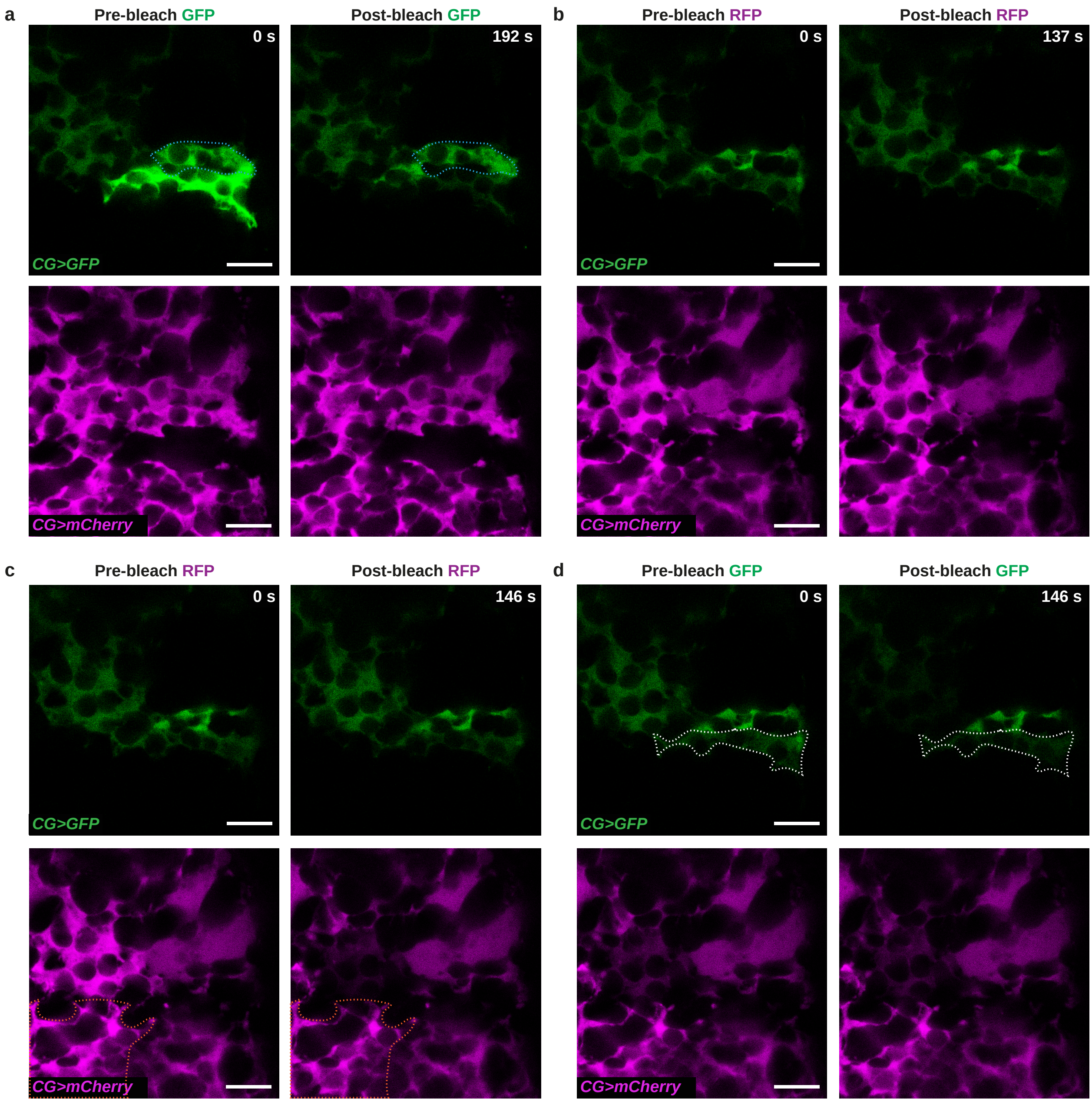

Rujano et al. Supplementary Figure 8

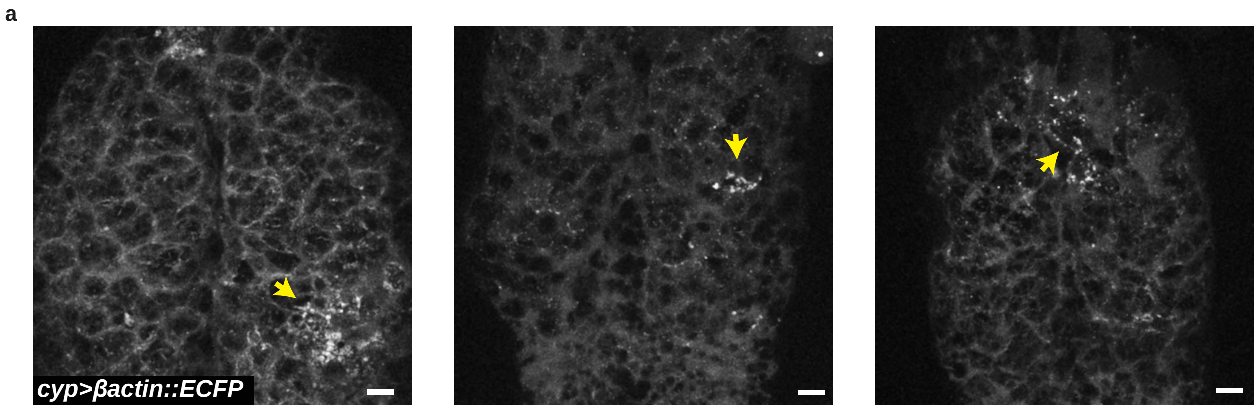

Rujano et al. Supplementary Figure 9

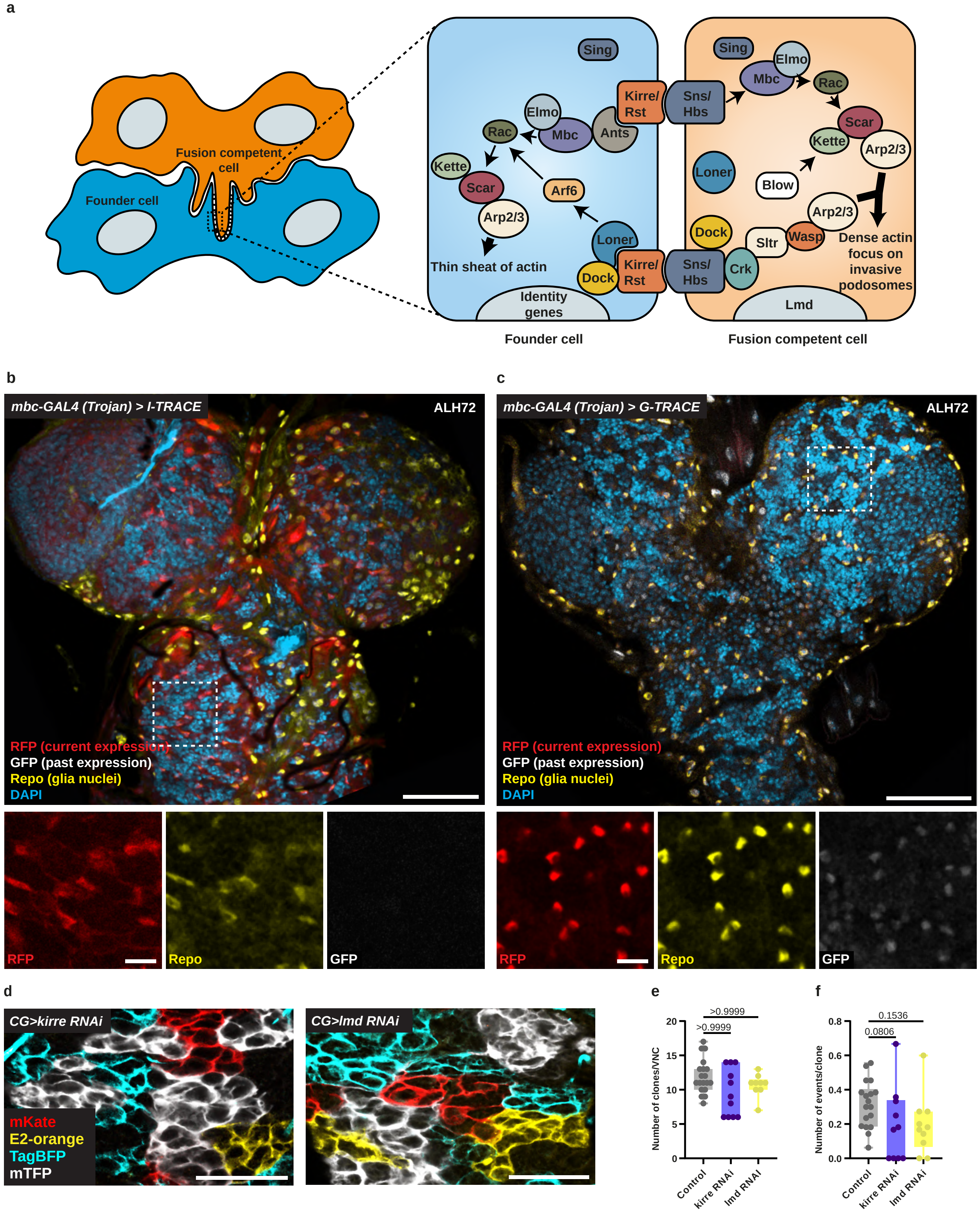

Rujano et al. Supplementary Figure 10

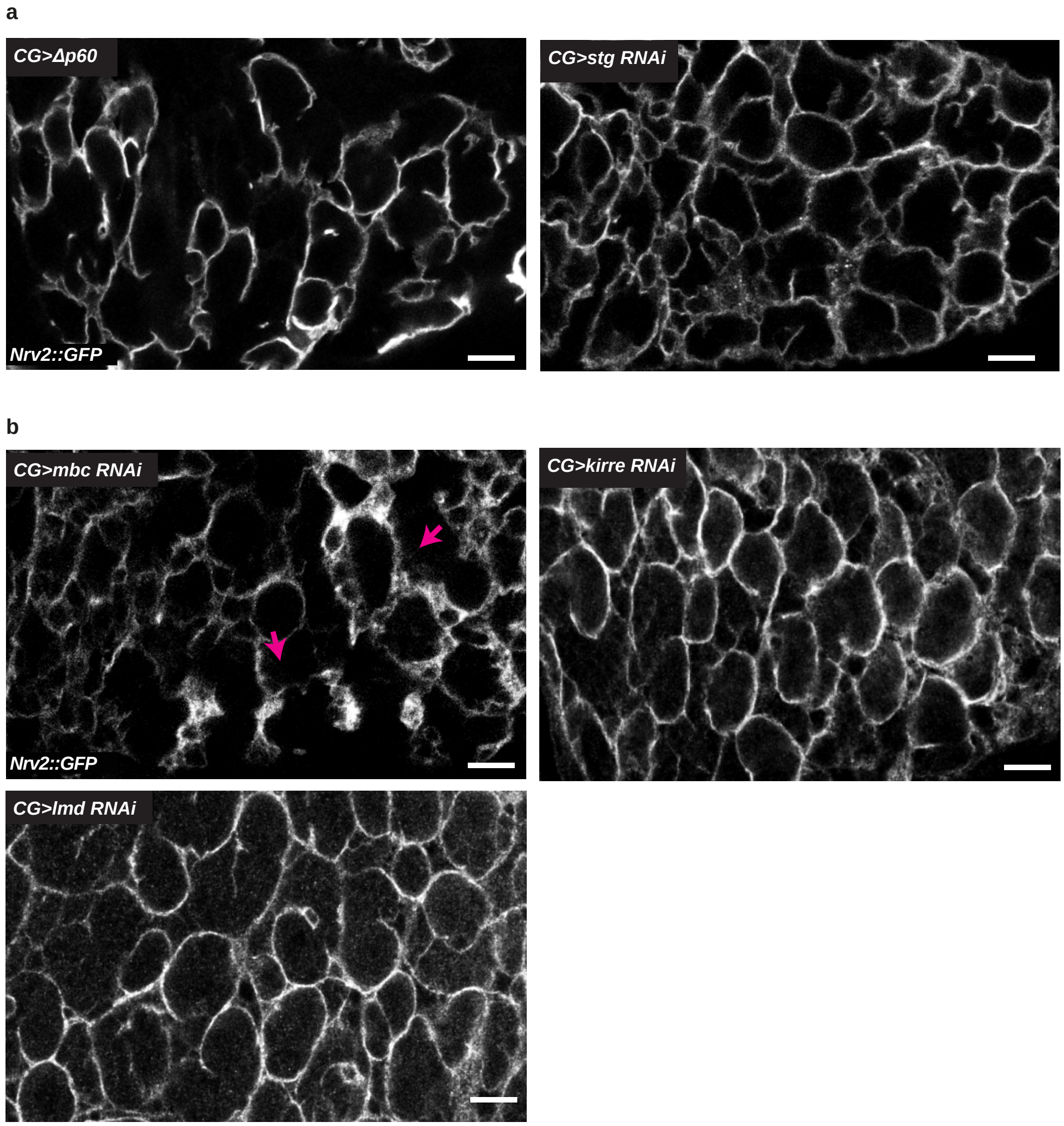

Supplement: Supplementary file 1 — Supplementary information [file 41467_2022_32685_MOESM1_ESM.pdf]
