# Supplementary material for: An interplay between cellular growth and atypical fusion defines morphogenesis of a modular glial niche in Drosophila
Source: Nat Commun. 2022 Aug 25;13:4999. doi: 10.1038/s41467-022-32685-3 (PMC9411534; doi:10.1038/s41467-022-32685-3)
Supplement: Supplementary file 17 — Reporting Summary [file 41467_2022_32685_MOESM17_ESM.pdf]

nature portfolio

Corresponding author(s): Pauline Spéder

Last updated by author(s): 2022/07/30

Reporting Summary

Nature Portfolio wishes to improve the reproducibility of the work that we publish. This form provides structure for consistency and transparency in reporting. For further information on Nature Portfolio policies, see our Editorial Policies and the Editorial Policy Checklist.

Statistics

For all statistical analyses, confirm that the following items are present in the figure legend, table legend, main text, or Methods section.

n/a	Confirmed	
☐	☒	The exact sample size (*n*) for each experimental group/condition, given as a discrete number and unit of measurement
☐	☒	A statement on whether measurements were taken from distinct samples or whether the same sample was measured repeatedly
☐	☒	The statistical test(s) used AND whether they are one- or two-sided<br>*Only common tests should be described solely by name; describe more complex techniques in the Methods section.*
☒	☐	A description of all covariates tested
☐	☒	A description of any assumptions or corrections, such as tests of normality and adjustment for multiple comparisons
☐	☒	A full description of the statistical parameters including central tendency (e.g. means) or other basic estimates (e.g. regression coefficient) AND variation (e.g. standard deviation) or associated estimates of uncertainty (e.g. confidence intervals)
☐	☒	For null hypothesis testing, the test statistic (e.g. *F*, *t*, *r*) with confidence intervals, effect sizes, degrees of freedom and *P* value noted<br>*Give P values as exact values whenever suitable.*
☒	☐	For Bayesian analysis, information on the choice of priors and Markov chain Monte Carlo settings
☒	☐	For hierarchical and complex designs, identification of the appropriate level for tests and full reporting of outcomes
☒	☐	Estimates of effect sizes (e.g. Cohen's *d*, Pearson's *r*), indicating how they were calculated

Our web collection on statistics for biologists contains articles on many of the points above.

Software and code

Policy information about availability of computer code

Data collection	Zeiss Zen software (2012 S4) was used for confocal acquisition of fixed and live samples from Drosophila central nervous system.
Data analysis	ImageJ/Fiji (2020 2.1.0/1,53c), Icy (2.2.1.0) and Volocity (6.3) were used to analyse confocal data.<br>Excel (16.53) was used to report manual counts and calculate ratios or percentages.<br>GraphPad Prism software was used for all statistical analyses (version 2021 9.0.1 (128)) except for:<br>1. Quantification of Fluorescence loss in photobleaching (FLIP): a Python script was written to perform a Monte-Carlo analysis and subsequently generate a Cumulative Distribution in order to calculate the value of percentage of fluorescence loss required for a 95% confidence level (for each fluorophore). The python script of this analysis was written with Python 3.6.15 , numpy 1.17.3 and matplotlib 2.2.5, and is available on Zenodo, DOI: 10.5281/zenodo.6941645.<br>2. Quantification of individual NSC ensheathing: a R script was written to calculate the significance of each condition compared to control using a generalized linear model (Binomial regression with a Bernoulli distribution).<br>Adobe Illustrator 25.4.6 and Photoshop 22.5.8 (both Adobe Creative Cloud) were used to assemble pictures and create figures.

For manuscripts utilizing custom algorithms or software that are central to the research but not yet described in published literature, software must be made available to editors and reviewers. We strongly encourage code deposition in a community repository (e.g. GitHub). See the Nature Portfolio guidelines for submitting code & software for further information.

Data

Policy information about availability of data

All manuscripts must include a data availability statement. This statement should provide the following information, where applicable:

- Accession codes, unique identifiers, or web links for publicly available datasets
- A description of any restrictions on data availability
- For clinical datasets or third party data, please ensure that the statement adheres to our policy

The datasets generated and/or analysed during the current study are available from the corresponding author upon reasonable request. The Python script used to calculate the significance of fluorescence loss during FLIP is available on Zenodo, DOI: 10.5281/zenodo.6941646

Field-specific reporting

Please select the one below that is the best fit for your research. If you are not sure, read the appropriate sections before making your selection.

- [x] Life sciences
- [ ] Behavioural & social sciences
- [ ] Ecological, evolutionary & environmental sciences

For a reference copy of the document with all sections, see nature.com/documents/nr-reporting-summary-flat.pdf

Life sciences study design

All studies must disclose on these points even when the disclosure is negative.

Sample size	No sample-size calculations were performed a priori. Sample sizes were determined by the number of processed samples (around 5-8 precisely staged dissected brains per experiment in most cases) and the number of independent experiments (mainly 2 to 3). This is routinely what we use in the lab for experimental design, with low variability between replicates observed from many experiments over the years (being partly due to the robustness of Drosophila genetics). See Spéder and Vrand 2018, and Benmimoun et al, 2020 as examples.
Data exclusions	No data were excluded, except rare outliers detected through the ROUT method (Q = 0.1%, GraphPad Prism 7.0a).
Replication	All replications were successful. Most experiments have been performed at least 2 times, except for except for Fig. 2b at ALH0; 3k; 5c; 6 (3 times, see main text); Supp. 2b,d and Supp. 5a-b at ALH24. For the FLIP experiments of Fig. 6, while the experimental procedure has been repeated three times, the results are not poolable due to the uniqueness of each situation (e.g., size and color labelling of CG units or extent of homotypic fusion).
Randomization	Randomization of larvae into groups was not possible most of the times as we were dealing with different genotypes that could not be mixed during the experimental procedure without loosing the ability to distinguish them easily, while they had to be tracked to assign a phenotype to a specific genetic manipulation (for exemple different RNAi lines driven by the same GAL4 line). For experiments looking at the change of a parameter over time, the difference in size of the animals/central nervous systems was enough to identify the stage and thus randomization was not relevant.
Blinding	Experimentators were not blinded, as most experiments required to prepare and keep separated different genotypes, and the experimentator could not be assisted by other members of the lab during the experimental procedures. However subsequent analysis by co-investigators was unbiased. For example, images were shown to another investigator without labelling/indicating the condition.

Reporting for specific materials, systems and methods

We require information from authors about some types of materials, experimental systems and methods used in many studies. Here, indicate whether each material, system or method listed is relevant to your study. If you are not sure if a list item applies to your research, read the appropriate section before selecting a response.

Materials & experimental systems

n/a	Involved in the study	
[ ]	[x]	Antibodies
[x]	[ ]	Eukaryotic cell lines
[x]	[ ]	Palaeontology and archaeology
[ ]	[x]	Animals and other organisms
[x]	[ ]	Human research participants
[x]	[ ]	Clinical data
[x]	[ ]	Dual use research of concern

Methods

n/a	Involved in the study	
[x]	[ ]	ChIP-seq
[x]	[ ]	Flow cytometry
[x]	[ ]	MRI-based neuroimaging

Antibodies

Antibodies used

1. PRIMARY
Guinea pig anti-Dpn (1:5000, in-house made, using pET29a-Dpn plasmid from J. Skeath for production)
Rabbit anti-Dpn (1:200, gift from R. Basto)
Chicken anti-GFP (1:2000, Abcam ab13970)
Rat anti-ELAV (1:100, DSHB 7E8A10-c)
Mouse anti-Repo (1:100, DSHB 8D12-c)
Rabbit anti-Phospho-histone H3 (1:100, Millipore 06-570)
Rat anti-mbc (1/200, gift from S. Abmayr)
Guinea pig anti-kirre (1/1000, gift from S. Abmayr)
Rabbit anti-Mucin D (1/1000, gift from AA. Kramerov).
Rabbit anti-Dcp1 (1/100, Cell Signalling 9578S)
2. SECONDARY
All secondary antibodies listed below were used at 1:200 dilution.
Goat anti-Chicken IgY (H+L) Secondary Antibody, Alexa Fluor 488 ThermoFisher Scientific A11039 2079383
Goat anti-Rabbit IgG (H+L) Secondary Antibody, Alexa Fluor 405 ThermoFisher Scientific A31556 (2249029)
Goat anti-Rabbit IgG (H+L) Highly Cross-Adsorbed Secondary Antibody, Alexa Fluor 488 ThermoFisher Scientific A11034 1971418
Goat anti-Rabbit IgG (H+L) Highly Cross-Adsorbed Secondary Antibody, Alexa Fluor 546 ThermoFisher Scientific A11035 2129899
Goat anti-Rabbit IgG (H+L) Highly Cross-Adsorbed Secondary Antibody, Alexa Fluor 633 ThermoFisher Scientific A21071 1932492
Goat anti-Guinea Pig IgG (H+L) Highly Cross-Adsorbed Secondary Antibody, Alexa Fluor 633 ThermoFisher Scientific A21105 2045328
Goat anti-Guinea Pig IgG (H+L) Highly Cross-Adsorbed Secondary Antibody, Alexa Fluor 488 ThermoFisher Scientific A11073 1637243
Goat anti-Rat IgG (H+L) Cross-Adsorbed Secondary Antibody, Alexa Fluor 488 ThermoFisher Scientific A11006 (34745A)
Goat anti-Rat IgG (H+L) Cross-Adsorbed Secondary Antibody, Alexa Fluor 546 ThermoFisher Scientific A11081 (2045302)
Goat anti-Rabbit IgG (H+L) Highly Cross-Adsorbed Secondary Antibody, Alexa Fluor 633 ThermoFisher Scientific A21071 1932492
Goat anti-Guinea Pig IgG (H+L) Highly Cross-Adsorbed Secondary Antibody, Alexa Fluor 633 ThermoFisher Scientific A21105 2045328
Goat anti-Guinea Pig IgG (H+L) Highly Cross-Adsorbed Secondary Antibody, Alexa Fluor 488 ThermoFisher Scientific A11073 1637243
Goat anti-Mouse IgG (H+L) Cross-Adsorbed Secondary Antibody, Alexa Fluor 546 ThermoFisher Scientific A11030 (1661231)

Validation

All commercial primary antibodies have been validated by suppliers as follows:
• chicken anti-GFP Abcam ab13970 (GR3190550-10). "The Abpromise guarantee covers the use of ab13970 for IHC-P, WB, ICC/IF, IHC-Fr, IHC-FoFr. The ab13970 antibody does cross-react with the many fluorescent proteins that are derived from the jellyfish Aequorea victoria. These are all proteins that differ from the original GFP by just a few point mutations (EGFP, YFP, mVenus, CFP, BFP etc.)."
• rabbit anti-phospho-Histone H3 (Ser10) Millipore 06-570 (3237504). Immunocytochemistry Analysis: Validation in HeLa and A431 cells. Immunoprecipitation Analysis: immunoprecipitated phospho Histone H3 (Ser10) from HeLa acid extract and Colcemid treated HeLa acid extracted RIPA lysate. Beadlyte® Assay Analysis: recognizes Histone H3 phosphorylated on Ser10 by Luminex assay.
• rat anti-ElaV 7E8A10 from DSHB: validated in the initial publication (Rubin GM, Cell 78.1 (1994 Jul 15): 137-47.)
• mouse anti-Repo 8D12 from DSHB: validated in the initial publication (Jones BW, Developmental biology 248.2 (2002 Aug 15): 369-83.)
• rabbit anti-Dcp-1 from Cell Signalling: validated by Western-Blot ("Cleaved Drosophila Dcp-1 (Asp215) Antibody recognizes endogenous levels of the large 22 kDa fragment of cleaved Dcp-1. This antibody does not recognize full length Dcp-1. The antibody also detects a non-specific, apoptotic-related band at 50 kDa by western blot.")

Antibodies received as gifts from individual labs, and not available commercially, were validated in previous studies :
• Rat anti-mbc: Erickson, M. R. S., Galletta, B. J. & Abmayr, S. M., J. Cell Biol. 138, 589–603 (1997)
• Guinea pig anti-Kirre: Galletta, B. J., Chakravarti, M., Banerjee, R. & Abmayr, S. M. Mech. Dev. 121, 1455–1468 (2004).
• Rabbi anti-Mucin D: Kramerov, A.A., Arbatsky, N.P., Rozovsky, Y.M., Mikhaleva, E.A., Polesskaya, O.O., Gvozdev, V.A., Shibaev, V.N. FEBS Lett. 378, 213–218 (1996).

Homemade antibodies:
• Guinea Pig anti-Dpn: it was validated using immunohistochemistry on Drosophila central nervous systems, showing it was specifically staining neural stem cells, and was co-staining with a previously published anti-Dpn antibody (from Jim Skeath).

Animals and other organisms

Policy information about studies involving animals; ARRIVE guidelines recommended for reporting animal research

Laboratory animals

A mix of Drosophila melanogaster male and female larvae were used for the experiments. Larvae were from different developmental ages (0, 24, 48, 72 and 96h at 25°C or 29°C), and were reared on standard cornmeal food.
The strains used are the following:
w1118 (BDSC 5905); Nervana2::GFP (Nrv2::GFP, BDSC 6828); tubulin-GAL80thermosensitive(ts) (BDSC 65406), Cre recombinase (BDSC 851), yw, hs-FLP (Andrea Brand lab), CoinFLP (BDSC 58750), cyp4g15-GAL4 (BDSC 39103), cyp4g15-FRT-STOP-FRT-LexA (this study), cyp4g15-FLP (this study), cyp4g1(-QF2 (this study), cyp4g15-mtd::Tomato (this study), alrm-GAL4 (Marc Freeman lab, Doherty et al ., 2009), mbc-GAL4 (Trojan, BDSC 66840), UAS-H2B::YFP (François Schweisguth lab, Bellaïche et al., 2001), UAS-H2B::RFP (Yohanns Bellaïche lab, Langevin et al, 2005), UAS-His3.3.mIFP-T2A-HO1 (BDSC 64184), UAS-GFP (BDSC 1522), UAS-mCD8::GFP (BDSC 5130), UAS-mCD8::RFP (BDSC 27399), UAS-mito::GFP (BDSC 8443), UAS-GFP::Alix (Jean-René Huynh lab, Eikenes et al., 2015), UAS-hßactin::ECFP (BDSC 7064), LexAOp-mCherry::mito (BDSC 66531), QUAS-NLS-LacZ (BDSC 3006), UAS-Raeppli CAAX 43E (BDSC 55082, this study), UAS-Raeppli NLS 53D (BDSC 55087, this study), LexAop-Raeppli CAAX 43E (BDSC 55082, this study), UAS-

	mRFP::Scra (BDSC 52220), Fly FUCCI (BDSC 55117), G-TRACE (BDSC 28280), iTRACE (BDSC 66387), Ubi-p63E-GFP::Pavarotti (David Glover lab, Minestrini et al., 2002), UAS-mbc RNAi (BDSC 32355), UAS-WASp RNAI (BDSC 51802), UAS-rst RNAi (VDRC 27223), UAS-hbs RNAi (BDSC 57003), UAS-kirre RNAi (VDRC 27227), UAS-lmd RNAi (BDSC 42871), UAS-sns RNAi (BDSC 64872), UAS-dock RNAi (BDSC 27728), UAS-dup RNAi (BDSC 29562), UAS-stg RNAi (BDSC 34831), UAS-Kaede (BDSC 26161), UAS-deltap60 (Weinkove et al., 1999)
Wild animals	No wild animals were used in this study.
Field-collected samples	No field-collected samples were used in this study.
Ethics oversight	Not relevant for Drosophila, which is not subjected to lab animal regulatory rules. For transgenics use and generation we have GMO approvals from the French Government (2016-2263 and 2017-2263bis).

Note that full information on the approval of the study protocol must also be provided in the manuscript.
